# A23 INTESTINAL STEM CELL REGULATION BY THE BACTERIAL METABOLITE ADP-HEPTOSE VIA ACTIVATION OF THE ALPK1-TIFA SIGNALLING PATHWAY

**DOI:** 10.1093/jcag/gwac036.023

**Published:** 2023-03-07

**Authors:** S Goyal, S Gray-Owen, S Girardin

**Affiliations:** 1 LMP; 2 MolGen, University of Toronto, Toronto, Canada

## Abstract

NOT PUBLISHED AT AUTHOR’S REQUEST

**Please acknowledge all funding agencies by checking the applicable boxes below:**

CCC, CIHR

**Disclosure of Interest:**

None Declared

